# Lipoprotein(a) and Its Autoantibodies in Association with Calcific Aortic Valve Stenosis

**DOI:** 10.3390/diseases11010043

**Published:** 2023-03-03

**Authors:** Anna L. Burdeynaya, Olga I. Afanasieva, Marat V. Ezhov, Elena A. Klesareva, Marina A. Saidova, Sergey N. Pokrovsky

**Affiliations:** 1 Laboratory of Lipid Disorders, Department of Atherosclerosis, A.L. Myasnikov Institute of Clinical Cardiology, Federal State Budgetary Institution National Medical Research Center of Cardiology Named after Academician E.I. Chazov, Ministry of Health of the Russian Federation, 121552 Moscow, Russia; 2 Laboratory of Atherosclerosis, Institute of Experimental Cardiology Named after Academician V.N. Smirnov, Federal State Budgetary Institution National Medical Research Center of Cardiology Named after Academician E.I. Chazov, Ministry of Health of the Russian Federation, 121552 Moscow, Russia; 3 Department of Ultrasound Diagnostics, A.L. Myasnikov Institute of Clinical Cardiology, Federal State Budgetary Institution National Medical Research Center of Cardiology Named after Academician E.I. Chazov, Ministry of Health of the Russian Federation, 121552 Moscow, Russia

**Keywords:** calcific aortic valve stenosis, lipoprotein(a), autoantibodies, coronary heart disease

## Abstract

Aortic valve stenosis is the most common valvular heart disease in the Western world. Lipoprotein(a) (Lp(a)) is an independent risk factor of coronary heart disease (CHD) and calcific aortic valve stenosis (CAVS). The aim of this study was to assess the role of Lp(a) and its autoantibodies [autoAbs] in CAVS in patients with and without CHD. We included 250 patients (mean age 69 ± 3 years, males 42%) and divided them into three groups. There were two groups of patients with CAVS depending on the presence (group 1) or absence of CHD (group 2). The control group included the patients without CHD or CAVS. According to logistic regression analysis, levels of Lp(a), IgM autoAbs to oxidized Lp(a) (oxLp(a)), and age were independent predictors of CAVS. A concomitant increase in Lp(a) level (≥30 mg/dL) and a decrease in IgM autoAbs concentration (<9.9 lab. Units) are associated with CAVS with an odds ratio (OR) of 6.4, *p* < 0.01, and with CAVS and CHD with an OR of 17.3, *p* < 0.001. IgM autoantibodies to oxLp(a) are associated with calcific aortic valve stenosis regardless of Lp(a) concentration and other risk factors. Higher Lp(a) and lower IgM autoantibodies to oxLp(a) levels are associated with a much higher risk of calcific aortic valve stenosis.

## 1. Introduction

Calcific aortic valve stenosis (CAVS) is the most common valve disease. Due to the aging of the population, CAVS may become a serious health burden in the next decade [1]. Male gender, hyperlipidemia (especially low-density lipoproteins (LDL) and lipoprotein(a) [Lp(a)]), arterial hypertension, smoking, type 2 diabetes and obesity are all risk factors that play a role in the early stages of aortic valve stenosis formation [2]. These risk factors lead to local inflammation and later to sclerosis and calcification of aortic valve leaflets [3]. In a large population-based study of more than half a million subjects in the USA, the prevalence of Lp(a) > 30 and >50 mg/dL were 35% and 24%, respectively [4]. A genomic study by the international consortium of the Cohorts for Heart and Aging Research in Genome Epidemiology (CHARGE) revealed a significant relationship between variation in the *LPA* gene encoding apolipoprotein(a) synthesis and the development and progression of CAVS [5].

There is evidence that systemic inflammation, as well as innate and adaptive immunity, plays a substantial role in the development and progression of atherosclerosis [6]. The oxidation-specific epitopes are found on oxidized lipoproteins, apoptotic cells, and extracellular vesicles. They are recognized by cellular pattern recognition receptors of innate immunity, such as C-reactive protein, complement factor H, and natural antibodies. [7]. The main biological functions of natural IgM antibodies are the removal of apoptotic cells, protection from infection, and maintenance of tissue homeostasis [8]. Our previous studies have shown that IgM autoantibodies (autoAbs) to native Lp(a) lower the risk of developing peripheral atherosclerosis [9]. The aim of this study was to assess the association of Lp(a), IgM autoAbs to native Lp(a), and oxidized Lp(a) (oxLp(a)), and CAVS in patients with and without CHD.

## 2. Material and Methods

This cross-sectional, single-center study included 250 patients aged over 45 years. The patients were divided into three groups according to the presence of CHD and CAVS. Patients who had CAVS and atherosclerotic diameter stenosis > 50% in at least one coronary artery according to angiography assessment constituted group 1 (n = 102, 41%). Group 2 consisted of 62 (25%) patients with CAVS without CHD. CHD was diagnosed based on coronary angiography results only. The third, or control, group had 86 (34%) subjects with intact or minimally affected coronary arteries, confirmed by coronary angiography, and an unaffected aortic valve, confirmed by transthoracic two-dimensional and Doppler echocardiography. Coronary angiography was performed in the control group due to nonspecific chest pain and the impossibility to exclude coronary artery disease by other methods. Exclusion criteria were congenital bicuspid aortic valve, rheumatic heart disease, infective endocarditis of the aortic valve, and any oncology or systemic connective tissue diseases. We included only patients with valvular aortic stenosis, excluding those with sub- and supravalvular aortic stenosis.

The study was conducted in accordance with the Helsinki Declaration and written informed consent was obtained from all patients. The local ethics committee (protocol No. 200 dated 27 October 2014) approved the study.

All patients underwent transthoracic two-dimensional and Doppler echocardiography to determine the parameters characterizing the functional state of the aortic valve. The diagnosis of aortic valve stenosis was established when the maximum blood flow velocity was >2.0 m/s and the average pressure gradient from the aortic valve was >20 mm Hg, according to the ACC/AHA guidelines for the management of patients with valvular heart disease [10]. We included patients with different grades of aortic valve stenosis: mild (21%), moderate (19%), severe (34%), and very severe (26%).

Routine blood tests to determine the concentration of total cholesterol (TC), triglycerides (TG), and high-density lipoprotein cholesterol (HDL-C) were performed with commercial kits (Biocon, Germany). The concentration of low-density lipoprotein cholesterol (LDL-C) was calculated using the Friedewald formula: LDL-C = TC − HDL-C − TG/2.2 (mmol/L), the level of LDL-C, corrected for Lp(a) cholesterol (LDL-C_corr_) was calculated as LDL_corr_ = LDL-C − 0.3 × Lp(a)/38.7 (mmol/L) [11]. The concentration of Lp(a) was measured using an enzyme linked immunosorbent assay with sheep monospecific polyclonal antibodies against human Lp(a) validated against commercial kits [12]. The level of specific autoaAbs against Lp(a) was determined by enzyme-linked immunosorbent assay according to a previously described method [13] with some modifications. For analysis, highly purified Lp(a) was coupled per 96 well EIA plate at a rate of 5 μg of Lp(a) per well. Tested samples of the patient’s serum were diluted in phosphate buffer containing 0.1% albumin and 0.05% Tween-20 in a ratio of 1:20. Anti-human IgM goat immunoglobulins conjugated with horseradish peroxidase were used for detecting antibodies. Registration of the reaction was carried out using orthophenyldiamine, measuring the absorption at a wavelength of 492 nm on an optical analyzer, Anthos 2010 Standard (Biochrom, Cambridge, UK). The serum of a healthy donor was used as a positive control, and physiological saline diluted similarly to the tested samples was used as a negative control.

Statistical analysis was performed using the MedCalc package (MedCalc, Mariakerke, Belgium). Normally distributed variables are presented as means with standard deviation; non-normally distributed variables are presented as medians and the 25th and 75th percentiles. The Kolmogorov-Smirnov test was used to determine the distribution normality. The Fisher’s exact test and the χ^2^ method were used to compare frequency scores between groups. Differences were considered statistically significant at *p* < 0.05. Threshold values of various markers were calculated by the receiver operative characteristics analysis (ROC analysis). The association of increased Lp(a) concentration with aortic valve stenosis in the general cohort and in different patient groups was analyzed by four-field tables to calculate the ORs and 95% confidence intervals (CI). To assess the analyzed parameters as independent factors associated with aortic valve stenosis, we performed multivariate logistic regression analysis with the inclusion of classical risk factors such as gender, age, smoking, hypertension, LDL-C, and diabetes in the model.

## 3. Results

The characteristics of the groups are presented in Table 1. Patients with aortic valve stenosis, regardless of CHD presence, were older than control subjects (*p* < 0.01). There were more males and people with type 2 diabetes in the first group. The majority of patients with CHD were on statins, patients with CAVS without CHD took statins more often than controls (*p* < 0.01). The levels of HDL-C and TG were comparable between the groups. The lowest concentration of TC and LDL-C was in patients in group 1, which is explained by more frequent use of statins in patients with CHD compared to the control group: 87% vs. 37%, *p* < 0.05.

There were more patients with severe aortic valve stenosis in group 2 than group 1. Maximum blood flow velocity through the aortic valve and the mean aortic valve pressure gradient were higher at 5 [4.; 5] vs. 4 [3; 5] m/s (*p* < 0.05), was 53 [40; 70] vs. 41 [24; 55] mm Hg (*p* < 0.05), respectively.

The concentration of Lp(a) in patients with CAVS, regardless of CHD presence, was higher than in controls. The Lp(a) level was highest in patients with CAVS and CHD (22.6 [6.5; 51.1] mg/dL) than in patients without CHD and controls: 14.0 [5.9; 48.3] and 12.1 [4.9; 25.1] mg/dL respectively, *p* < 0.05 for both.

**Figure 1:**
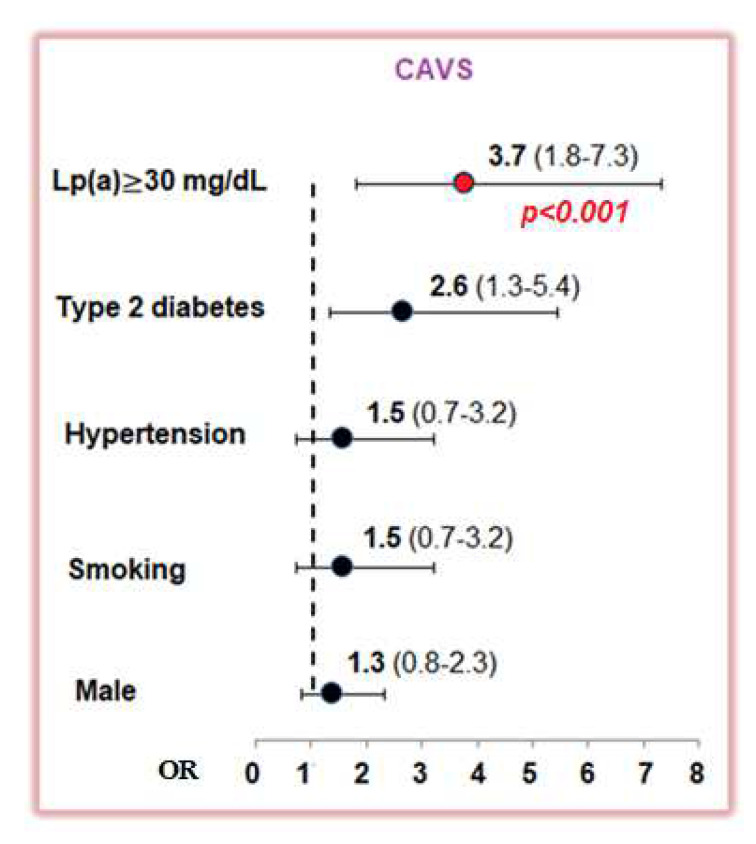
The odds ratio for calcific aortic valve stenosis according single-factor analysis for lipoprotein(a) ≥ 30 mg/dL and classic risk factors. CAVS—calcific aortic valve stenosis; Lp(a)—lipoprotein(a).

According to the ROC analysis, an Lp(a) concentration ≥ 30 mg/dL predicted the presence of aortic valve stenosis with a sensitivity of 39% and a specificity of 84%, area under the curve 0.63, *p* < 0.001 (Figure S1). In the overall cohort of examined patients, an elevated Lp(a) level (≥30 mg/dL) was associated with aortic valve stenosis with an OR of 3.7; 1.8–7.3, *p* < 0.001. Only type 2 diabetes was associated with the presence of aortic valve stenosis: OR 2.6 (1.3–5.4), *p* < 0.05 (Figure 1).

In the presence of an Lp(a) level above 30 mg/dL the OR for CAVS and CHD was 4.7 (2.1–10.2) and 3.4 for CAVS without CHD (1.4–8.0), *p* <0.05. In a model adjusted for age, gender, smoking, and LDL-C level, an Lp(a) ≥ 30 mg/dL with an OR of 3.63 (95% CI 1.47–8.98), *p* = 0.005 was associated with the presence of aortic valve stenosis in the logistic regression analysis.

We did not find an association between Lp(a) concentration and severity of aortic valve stenosis.

**Figure 2:**
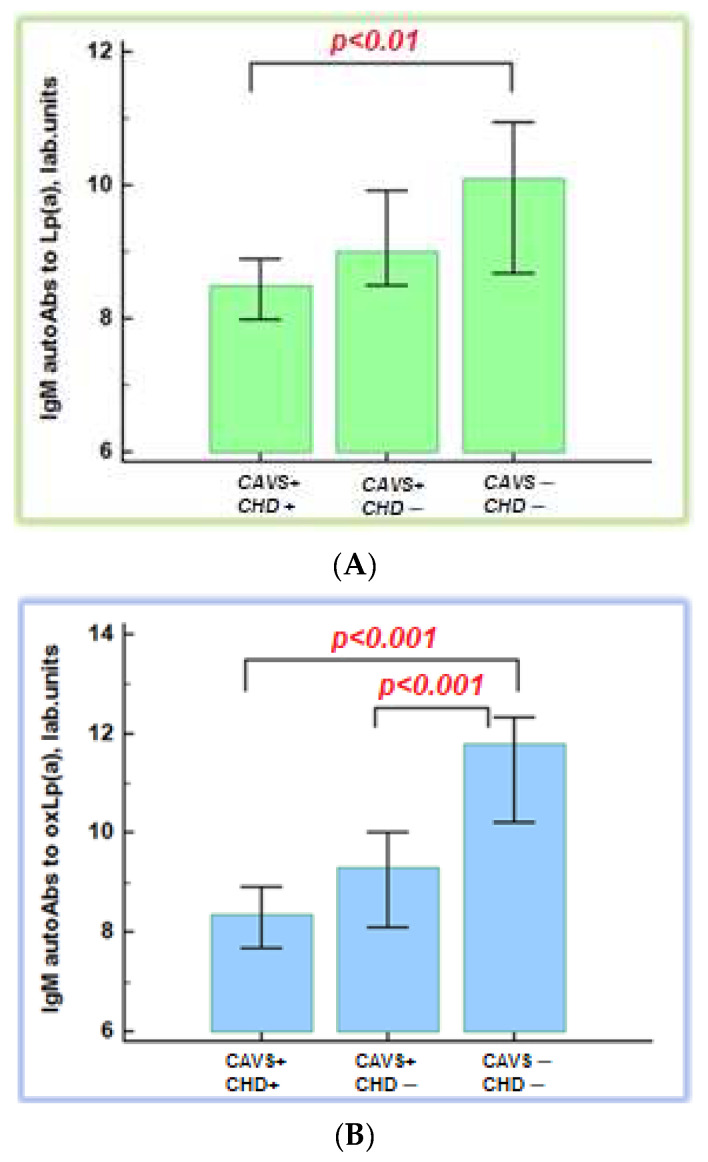
Immunoglobulin M autoantibodies to native (**A**) and oxidative lipoprotein(a) (**B**). CAVS—calcific aortic valve stenosis; CHD—coronary heart disease; autoAbs—autoantibodies; Lp(a)—lipoprotein(a); oxLp(a)—oxidized lipoprotein(a).

The maximal level of IgM autoAbs to Lp(a) and oxLp(a) was in group 3. The level of IgM autoAbs to Lp(a) was significantly lower in patients with CAVS and CHD compared to control subjects: 8.5 (7.1; 9.8) and 10.1 (8.1; 12.0) lab. units, respectively, *p* < 0.01. The level of autoAbs to oxLp(a) was significantly lower in patients with CAVS regardless of CHD presence (Figure 2). We found no association between maximal level of IgM autoAbs to oxLp(a) and the severity of aortic valve stenosis.

There were more patients with CAVS and CHD who had an Lp(a) level ≥ 30 mg/dL. The level of IgM autoAbs to oxLp(a) was higher in patients with an Lp(a) concentration < 30 mg/dL than in those with an Lp(a) concentration ≥ 30 mg/dL (9.7 (8.1; 10.6) versus 8.7 (7.7; 9.6) lab. units, respectively, *p* < 0.05)

According to logistic regression analysis, age (OR 1.15 (1.08–1.22)) and Lp(a) (OR 1.02 (1.01–1.04) were independently associated with CAVS, and increasing Lp(a) concentration by 1 mg/dL increased the probability of aortic valve stenosis by 2%. IgM autoAbs to oxLp(a) reduced the likelihood of the presence of CAVS: OR 0.81 (0.71–0.92), *p* < 0.05 for all (Table S1)

ROC-analysis showed that the cut-off value for IgM autoAbs to oxLp(a) is 9.9 lab. Units, which increases the likelihood of aortic valve stenosis with a sensitivity of 68% and a specificity of 71%, an area under the curve of 0.74, *p* < 0.001 (Figure S2).

**Figure 3:**
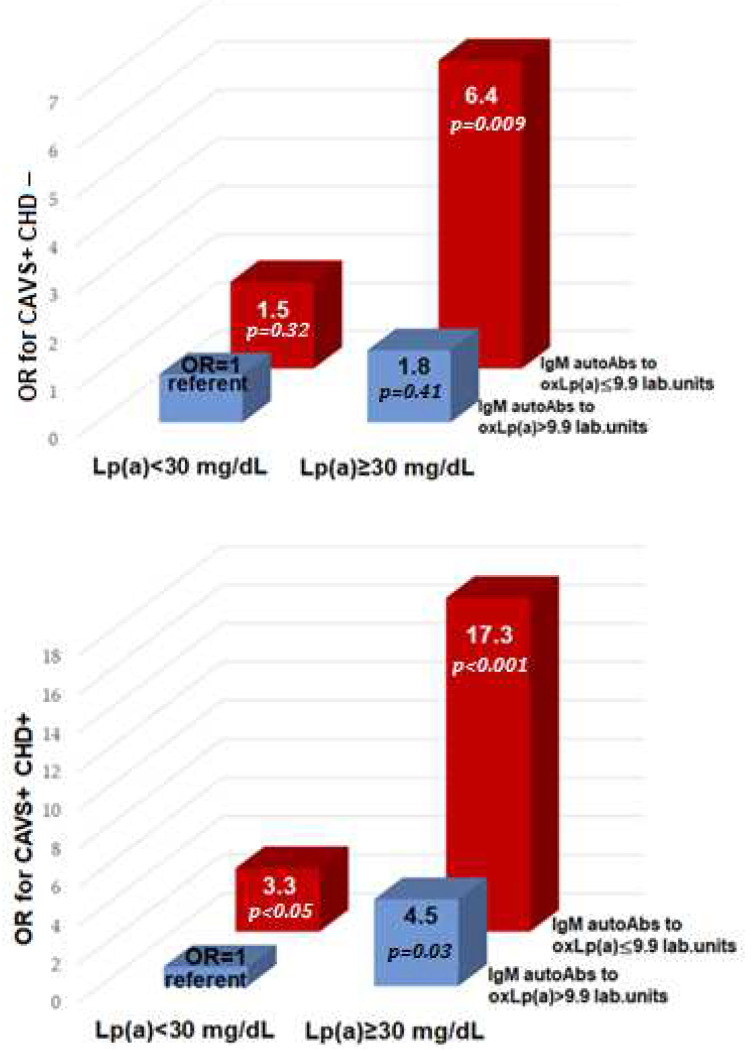
Odds ratio of calcific aortic valve stenosis and coronary heart disease depending on lipoprotein(a) and IgM autoantibodies to oxidative lipoprotein(a). OR—odds ratio; CAVS—calcific aortic valve stenosis; CHD—coronary heart disease; autoAbs—autoantibodies; Lp(a)—lipoprotein(a); oxLp(a)—oxidized lipoprotein(a).

An increase in Lp(a) ≥ 30 mg/dL and a decrease in IgM autoAbs less than 9.9 lab. units is associated with CAVS with and without CHD with an OR of 17.3 (4.2–90.1; *p* < 0.001) and 6.4 (1.8–43.1; *p* < 0.01), respectively (Figure 3).

## 4. Discussion

Each year, approximately 67,500 surgical aortic valve replacements are performed in the United States [14]. According to a large study including 1474 subjects with aortic valve stenosis, the mean age of patients who underwent aortic valve replacement was 77.6 years [15]. In our study, patients with aortic valve stenosis were significantly older than patients without aortic valve stenosis, regardless of the presence of CHD. Risk factors such as male gender, hyperlipidemia, arterial hypertension, smoking, and type 2 diabetes play a role in the development of aortic valve stenosis in association with age [2]. Arterial hypertension, obesity, and smoking were not associated with aortic valve stenosis in our study, most likely because the control group included patients who had similar occurrences of these factors in the absence of aortic valve stenosis and CHD. Type 2 diabetes is the only classic risk factor associated with aortic valve stenosis. Since 1995, researchers have been investigating Lp(a) as a potential risk factor for aortic valve stenosis. In a large prospective study such as Cancer and Nutrition (EPIC)-Norfolk, which included 17,553 participants, 118 patients developed aortic valve calcification over an 11.7-year follow up. When compared with participants with Lp(a) levels corresponding to the lower tertile (≤4.8 mg/dL), patients with Lp(a) levels corresponding to the upper tertile (>40.9 mg/dL) had a higher risk of aortic valve calcification (OR 1.57; 1.02–2.42) [16]. The results of our cross-sectional study confirm previous data on the role of elevated concentrations of Lp(a) in aortic valve stenosis. Thus, the average concentration of Lp(a) in patients with aortic valve stenosis was more than twice as high as in patients without aortic valve stenosis. The highest median level of Lp(a) was observed in patients with CAVS and CHD. According to our data, the odds ratio of aortic valve stenosis, without taking into account concomitant CHD, in the presence of a Lp(a) concentration greater than or equal to 30 mg/dL was 3.7 (1.8–7.3). The elevated Lp(a) level was associated with CAVS and CHD with an OR of 4.7 (2.1–10.2) and with CAVS without CHD with an OR of 3.4 (1.4–8.0). Notably, most studies examining the role of Lp(a) in aortic valve stenosis have not assessed the impact of concomitant CHD.

According to the Canadian Heart Society’s guidelines for dyslipidemia and the Apheresis Society of America [17,18], Lp(a) concentrations of greater than or equal to 30 mg/dL are associated with cardiovascular diseases. At the same time, according to the recommendation of the Board of the College of Cardiology and the Association of the Heart, as well as the European Society for the History of Atherosclerosis and Dyslipidemia, the threshold value of the accepted concentration of Lp(a) is 50 mg/dL [19,20]. The EPIC-Norfolk study showed that after adjusting for sex, age, smoking, and LDL-C, Lp(a) concentration ≥ 50 mg/dL was associated with aortic valve stenosis with an OR of 1.98; 1.25–3.09 compared with patients with Lp(a) levels < 50 mg/dL [16]. In the Aortic Stenosis Progression Monitoring: Measuring the Effects of Rosuvastatin (ASTRONOMER) study, an Lp(a) level corresponding to the upper tertile (>58.5 mg/dL) was an independent risk factor associated with rate of progression of aortic valve stenosis in patients with mild to moderate aortic valve stenosis. In our study, Lp(a) concentrations above 30 mg/dL were associated with the presence of aortic valve stenosis with a sensitivity of 39% and a specificity of 84%. After multivariable adjustment according to age, gender, smoking, and LDL-C level an Lp(a) level ≥ 30 mg/dL was associated with the presence of aortic valve stenosis. According to our results the frequency of detecting Lp(a) levels ≥ 30 mg/dL was significantly higher among patients with aortic valve stenosis, with an incidence of 43% and 34% in patients with concomitant CHD and without CHD, respectively, compared to the control group, where the incidence of hyperlipoproteinemia(a) was 12%. In a large study including 2710 patients with severe aortic valve stenosis, Lp(a) level was measured among 124 subjects, 33% of them had an Lp(a) concentration greater than or equal to 30 mg/dL [21].

It has been shown that CAVS does not develop because of age but is rather related to a complex pathophysiological process [22], a kind of inflammation under the influence of biochemical, genetic, and humoral factors [23]. The early stages of aortic valve stenosis have similar pathogenetic mechanisms to atherosclerosis [24]. Oxidation of LDL is one of the initiating factors in the development of atherosclerosis. This form of LDL leads to more intense damage of the vascular endothelium; in addition, oxidized regions of LDL, such as epitopes of malondialdehyde (MDA) and oxidized phospholipids (oxPL), are specific regions serving as a ligand for innate immunity cells, such as natural IgM antibodies [7,25]. These antibodies are produced by B-1 lymphocytes uced to protect the body from various pathogens. By binding to an oxidized epitope on the surface of LDL, IgM recognizes them as pathogenic organisms and eliminates them from the body, thereby preventing the progression of atherosclerosis. In previous studies, we evaluated the contribution of Lp(a) as a possible autoantigen in the development of atherosclerosis and determined the level of circulating autoAbs against Lp(a) [9,26]. In this study, we assessed the association of IgM autoAbs to Lp(a) and oxLp(a) with degenerative aortic valve stenosis. We found that the level of autoAbs to both native Lp(a) and oxLp(a) was highest in control subjects. The amount of IgM autoAbs to oxLp(a) was lower in patients with aortic valve stenosis, regardless of the presence of CHD, when compared with control subjects. The level of IgM to Lp(a) was lower in patients with aortic valve stenosis and CHD and did not differ significantly in patients with aortic valve stenosis without CHD compared with the control group. In a subanalysis of the ASTRONOMER study, which included 220 patients with mild to severe aortic valve stenosis, there was no difference in the rate of progression of aortic valve stenosis depending on the tertile levels of IgG and IgM autoantibodies to LDL modified with MDA-LDL and apoB-IC. However, risk analysis of rapid progression of aortic valve stenosis showed a trend for higher IgG against to ApoB-IC levels and the lowest tertile of IgM against to MDA-LDL [27]. Thus, an increase in IgM levels has a protective effect, reducing the likelihood of aortic valve stenosis and its progression. It should be noted that the ASTRONOMER sub-study was prospective and did not assess the relationship of these parameters with the early stages of aortic valve stenosis development. Whether these autoantibodies play a role in the initiation and development of CAVS remains to be determined in epidemiological studies in individuals with no pre-existing CAVS who are followed prospectively. In addition, in our study, we did not exclude patients with CHD, diabetes mellitus, and heart failure as the authors did in the ASTRONOMER sub-study. Similar to our study, patients with more progression of aortic valve stenosis were older than those with less progression [27]. A 15-year prospective study showed that a higher level of IgM autoAbs against MDA-LDL is associated with a lower risk of acute coronary syndrome and newly registered CVD [28]. We found that patients with an Lp(a) ≤ 30 mg/dL had higher levels of IgM autoantibodies to oxLp(a) than those with an Lp(a) > 30 mg/dL. According to logistic regression analysis, IgM autoantibodies to oxLp(a) with an OR of 0.81 (0.71–0.92) were a factor reducing the likelihood of having aortic valve stenosis, *p* < 0.05. These results may characterize the ability of IgM to bind to Lp(a), as a potential antigen, and eliminate it from the bloodstream, reducing the likelihood of developing aortic valve stenosis.

Several large randomized trials (SALTIRE, SEAS, and ASTRONOMER) have investigated the role of statins in the progression of aortic valve stenosis, but none of them have shown positive results [29,30,31]. One possible reason for this is that patients with moderate and severe aortic valve stenosis were included in these trials. In our opinion, drug therapy may be most effective in the early stages of the disease, during the inflammatory process, rather than during calcification. In a sub-study FOURIER, early use of evolocumab reduced the risk of aortic valve stenosis by the second year compared with placebo. The overall hazard ratio for aortic valve stenosis with evolocumab was 0.66 (0.40–1.09). There was no apparent association in the first year (hazard ratio: 1.09 (0.48–2.47)), but there was a hazard ratio of 0.48 (0.25–0.93) after the first year of treatment compared with placebo (*p* < 0.05) [32]. Several studies suggest that vaccination can be used to prevent atherosclerosis [33,34], but this direction requires further research. Our results may serve as a suggestion for prospective studies in this direction.

## 5. Study Limitations

Our study included a small cohort of patients, which limits the significance of the results and necessitates further, larger studies. Some differences were also found between the control group and the groups with aortic valve stenosis with and without CHD, including a significant difference in age. A multiple logistic regression analysis model showed the independence of the relationship of Lp(a), autoAbs against oxLp(a) from age and other risk factors for CAVS and CHD. Our study is also limited to the study of IgM autoAbs against Lp(a) and oxLp(a), without assessing the value of autoAbs of other types, such as autoAbs against MDA-LDL or oxLDL.

## 6. Conclusions

Lp(a) concentrations ≥ 30 mg/dL are associated with calcific aortic valve stenosis regardless the presence of coronary heart disease. IgM autoantibodies to oxLp(a) are related to calcific aortic valve stenosis regardless Lp(a) concentration and other risk factors. Concomitant higher Lp(a) concentration and lower IgM autoantibodies to oxLp(a) levels are associated with a significantly increased probability of calcific aortic valve stenosis.

## Figures and Tables

**Table 1:** General characteristics of patients.

Parameter	Group 1 CAVS with CHD n = 102	Group 2 CAVS, No CHD n = 62	Group 3 No CAVS, No CHD n = 86
Age, years	72 ± 11 *	74 ± 7 *	59 ± 13
Male sex	55 (53%) *^#^	18 (29%)	29 (34%)
Smoking	29 (28%)	8 (13%)	13 (15%)
Hypertension	93 (91%)	51 (82%)	64 (74%)
Type 2 diabetes	37 (36%) * ^#^	13 (20%)	11 (13%)
Total cholesterol, mmol/L	5.0 ± 2.2 *^#^	5.3 ± 1.6	5.6 ± 1.5
Triglycerides, mmol/L	1.5 ± 0.7	1.5 ± 0.6	1.7 ± 1.0
HDL-C, mmol/L	1.1 ± 0.3	1.3 ± 0.3	1.3 ± 0.4
LDL-C, mmol/L	3.2 ± 2.0 *^#^	3.4 ± 0.4	3.6 ± 1.4
Statin therapy	89 (87%) *^#^	40 (65%) *	32 (37%)
Antiaggregating therapy	92 (90%) *^#^	38 (61%) *	24 (28%)
Beta-blocker therapy	84 (82%) *	47 (76%) *	42 (49%)
Calcium channel blocker therapy	21 (21%)	12 (19%)	16 (19%)
ACE inhibitor therapy	49 (48%)	23 (36%)	30 (35%)
Severe aortic valve stenosis	52 (51%) ^#^	48 (77%)	0 (0%)
Vmax, m/s	4 [3; 5] ^#^	5 [4; 5]	n/a
MPG, mm Hg	41 [24; 55] ^#^	53 [40; 70]	n/a
TPG (max), mm Hg	63 [24;92]	77 [47; 116]	n/a

* *p* < 0.01 compared to control; ^#^ *p* < 0.05 group 1 vs. 2. Data are presented as an absolute number and percent of patients—n (%) or mean ± standard deviation. HDL-C, high-density lipoprotein cholesterol; LDL-C, low-density lipoprotein cholesterol; ACE inhibitors—angiotensin-converting enzyme inhibitors; Vmax—peak aortic jet velocity; MPG—mean aortic transvalvular pressure gradient; TPG (max)—maximal transvalvular pressure gradient.

## Data Availability

Data will be made available by the corresponding author upon reasonable request.

## Supplementary Materials

The following supporting information can be downloaded at: https://www.mdpi.com/article/10.3390/diseases11010043/s1, Figure S1: Multiple regression analysis of different parameters associated with aortic valve stenosis.; Figure S2: ROC curve analysis for lipoprotein(a); Table S1: ROC curve analysis for IgM autoantibodies to oxidised lipoprotein(a).
