# Supplementary material for: Lipoprotein(a) and Its Autoantibodies in Association with Calcific Aortic Valve Stenosis
Source: Diseases. 2023 Mar 3;11(1):43. doi: 10.3390/diseases11010043 (PMC10047835; doi:10.3390/diseases11010043)
Supplement: Supplementary file 1 [file diseases-11-00043-s001.zip › diseases-2238790-supplementary.pdf]

Table S1. Multiple regression analysis of different parameters associated with aortic valve stenosis.

Parameter	Model 1	Model 2	Model 3	Model 4	Model 5
Lipoprotein(a)	1.03 (1.01 - 1.04)	1.02 (1.00 - 1.03)	1.02 (1.00 - 1.03)	1.02 (1.00 - 1.04)	1.02 (1.00 - 1.04)
autoAbs IgM	-	0.87 (0.81 - 0.93)	0.88 (0.82- 0.95)	0.84 (0.76 - 0.92)	0.81 (0.71 - 0.92)
Age	-	-	1.11 (1.07 - 1.16)	1.11 (1.06 - 1.16)	1.15 (1.08 - 1.22)
Type 2 diabetes	-	-	-	1.82 (1.04 - 3.19)	1.95 (1.04 - 3.67)
AUC	0.68	0.74	0.85	0.88	0.87
Percent of cases correctly classified	68.31%	75.59%	82.16%	83.08%	79.58%

The logistic model included: Model 1 - Lipoprotein(a); Model 2 - Lipoprotein(a) and autoantibodies against oxidised Lipoprotein(a); Model 3 - Lipoprotein(a), autoantibodies against oxidised Lipoprotein(a) and age; Model 4 - Lipoprotein(a), autoantibodies against oxidised Lipoprotein(a), age and diabetes; Model 5 - all risk factors including sex, smoking, hypertension, body mass index, LDL-C levels.

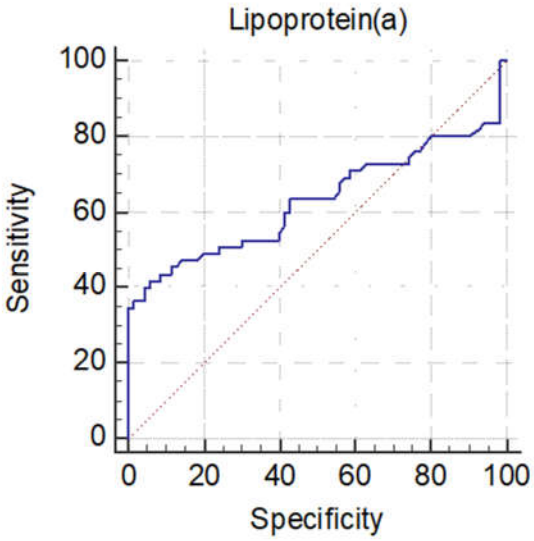

Figure S1. ROC curve analysis confirmed lipoprotein(a) serum level of 30 mg/dl as the best cut-off value for aortic valve stenosis with sensitivity – 39%, specificity – 84%, AUC – 0.63, $p<0.001$.

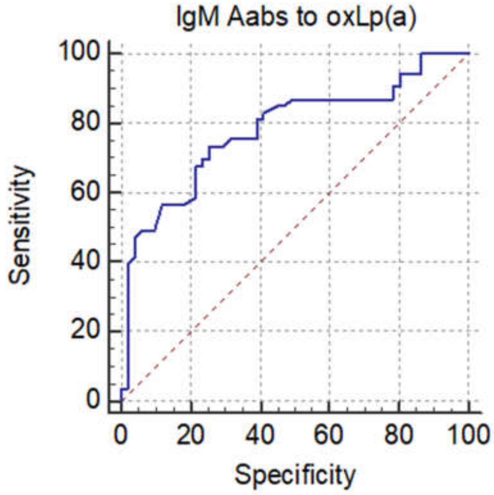

Figure S2. ROC curve analysis for IgM autoantibodies to oxidised lipoprotein(a) confirmed a level of 9.9 lab.units as the best cut-off value for aortic valve stenosis, sensitivity – 68%, specificity – 71%, AUC – 0.74, p<0.001.
